# Building a model for disease classification integration in oncology, an approach based on the national cancer institute thesaurus

**DOI:** 10.1186/s13326-017-0114-4

**Published:** 2017-02-07

**Authors:** Vianney Jouhet, Fleur Mougin, Bérénice Bréchat, Frantz Thiessard

**Affiliations:** 1 CHU de Bordeaux, Pole de sante publiqueService d’information medicale, unit IAM, F-33000Bordeaux, France; 2 Univ. Bordeaux, Inserm, UMR 1219, Bordeaux, F-33000 France

**Keywords:** NCI thesaurus, Oncology, Terminology, Semantic integration, ICD-10, ICD-O-3

## Abstract

**Background:**

Identifying incident cancer cases within a population remains essential for scientific research in oncology. Data produced within electronic health records can be useful for this purpose. Due to the multiplicity of providers, heterogeneous terminologies such as ICD-10 and ICD-O-3 are used for oncology diagnosis recording purpose. To enable disease identification based on these diagnoses, there is a need for integrating disease classifications in oncology. Our aim was to build a model integrating concepts involved in two disease classifications, namely ICD-10 (diagnosis) and ICD-O-3 (topography and morphology), despite their structural heterogeneity. Based on the NCIt, a “derivative” model for linking diagnosis and topography-morphology combinations was defined and built. ICD-O-3 and ICD-10 codes were then used to instantiate classes of the “derivative” model. Links between terminologies obtained through the model were then compared to mappings provided by the Surveillance, Epidemiology, and End Results (SEER) program.

**Results:**

The model integrated 42% of neoplasm ICD-10 codes (excluding metastasis), 98% of ICD-O-3 morphology codes (excluding metastasis) and 68% of ICD-O-3 topography codes. For every codes instantiating at least a class in the “derivative” model, comparison with SEER mappings reveals that all mappings were actually available in the model as a link between the corresponding codes.

**Conclusions:**

We have proposed a method to automatically build a model for integrating ICD-10 and ICD-O-3 based on the NCIt. The resulting “derivative” model is a machine understandable resource that enables an integrated view of these heterogeneous terminologies. The NCIt structure and the available relationships can help to bridge disease classifications taking into account their structural and granular heterogeneities. However, (i) inconsistencies exist within the NCIt leading to misclassifications in the “derivative” model, (ii) the “derivative” model only integrates a part of ICD-10 and ICD-O-3. The NCIt is not sufficient for integration purpose and further work based on other termino-ontological resources is needed in order to enrich the model and avoid identified inconsistencies.

**Electronic supplementary material:**

The online version of this article (doi:10.1186/s13326-017-0114-4) contains supplementary material, which is available to authorized users.

## Background

With the increasing adoption of electronic health records (EHRs), the amount of data produced at the patient bedside is rapidly increasing. These data provide new perspectives to: create and disseminate new knowledge; consider the implementation of personalized medicine and offer to patients the opportunity to be involved in the management of their own medical data [1]. Secondary use of biomedical data produced throughout patient care is an essential issue [2] and is the subject of numerous studies over several years [1–6]. Since 2007, the American Medical Informatics Association emphasized the value of secondary use of medical data: *“Secondary use of health data can enhance healthcare experiences for individuals, expand knowledge about disease and appropriate treatments, strengthen understanding about the effectiveness and efficiency of our healthcare systems, support public health and security goals, and aid businesses in meeting the needs of their customers”* [4].

In the oncology field, it is necessary to identify and describe incident cancer cases within a population in order to facilitate research and public health monitoring. For instance, cancer registries exhaustively record incident cases of cancer in a given territory (which correspond to all new cancer cases occurring over a geographical territory). This task remains time consuming if it is performed manually. As early as 1998, a technical report was drawn up by the International Agency for Research on Cancer describing the methods used by different registries for establishing automated procedures to identify new cases using available data [7]. Methods have been proposed for automatically identifying and registering cancers using structured data indexed with standard terminologies [8–12].

However, many different medical specialties are contributing to record information in EHRs. As a result, within EHRs, data describing diseases are recorded according to multiple heterogeneous terminologies even for a single disease occurring in a single patient. For instance, in France, reimbursement data use the 10th revision of the International Statistical Classification of Diseases and Related Health Problems (ICD-10) [13] to describe diseases, whereas pathology data use either ADICAP (a French pathology terminology) or the 3rd edition of the International Classification of Diseases for Oncology (ICD-O-3) [14] and data from multidisciplinary meetings in oncology use ICD-O-3. Providing an integrated access to these disease classifications may improve automated cancer identification.

Although ICD-10 and ICD-O-3 both describe cancer diseases, they exhibit differences in terms of structure and granularity. Thus, it is necessary to identify or to build a resource that allows the integration of cancer disease classifications, taking into account these heterogeneities. To achieve this goal, relations must be defined between the involved concepts, such as “a neoplasm is a disease and has a specified morphology as well as a specified topography”. The National Cancer Institute thesaurus (NCIt)*“provides reference terminology covering vocabulary for clinical care, translational and basic research, and public information activities”*(cited from http://ncit.nci.nih.gov/, visited 2015-01-22). It is described as *“a controlled terminology which exhibits ontology-like properties in its construction and use”* [15]. These characteristics *“open up the possibility [...] in linking together heterogeneous resources created by institutions external to the NCI”* [16]. Thus, the NCIt could be used as a resource to bridge the gap between disease classifications, which are structurally heterogeneous.

However, since 2005, it has been shown on many occasions that the NCIt remains flawed [16–18] and especially that logic-based reasoning over the NCIt should be used cautiously. On the other hand, re-building a model “from scratch” would be time consuming and comes with no guarantee of avoiding inconsistencies. Despite the limitations described above, the NCIt contains knowledge that could be useful for our integration purpose. In this manuscript, we propose an approach to build a resource based on a subset of the NCIt, linking the three axes that refer to diseases as described in ICD-10 and ICD-O-3, i.e., the diagnosis as well as its morphology and its topography.

### ICD-10

Within ICD-10, chapter 2 corresponds to neoplasms. It is divided into four axes depending on the behavior of the tumor (namely *Malignant neoplasms*, *In situ neoplasms*, *Benign neoplasms* and *Neoplasms of uncertain or unknown behavior*). Within the *Malignant neoplasms* block, ICD-10 categories differentiate primary tumors from metastatic secondary tumors. In the same way as for ICD-O-3, a neoplasm cannot have multiple behaviors. ICD-10 describes each neoplastic disease as a whole concept represented by a unique code. For instance, C50.2: *Malignant neoplasm upper-inner quadrant of breast* describes two characteristics of the cancer disease:

- The behavior (*Malignant*) which is part of the morphology description.
- The site of origin (*upper-inner quadrant of breast*) which corresponds to the topography.

### ICD-O-3

ICD-O-3 is a multi-axial classification used in cancer registries in order to record the anatomic site (topography) and the morphology of a neoplasm. The morphology is coded with five digits. The first four digits represent the histological description and the fifth digit indicates the behavior (i.e. whether benign or malignant) of a neoplasm. As a result, it is not possible for a morphology to have multiple behaviors. *“The topography code indicates the site of origin of a neoplasm; in other words, where the tumor arose”* [14]. From the ICD-O-3 “point of view”, any morphology code can be associated with any topography code. Some tumor morphologies have a *“usual primary site”* but it is expressly stated that these associations are provided only to help coders and should not be considered as systematic (and unique) topography-morphology combinations. An example is given in [14]: *“An unusual, but possible, example would be the diagnosis ’osteo-sarcoma of kidney’, for which the kidney topography code (C64.9) would be used instead of ’bone, NOS’ (C41.9) […]”*. Thus, ICD-O-3 describes a disease by combining the morphology of the tumor and the topography from where the tumor arises. As a result, each neoplastic disease is not described as a whole concept entailed by a unique code within ICD-O-3.

### Concepts involved in ICD-10 and/or ICD-O-3

Even if they are called “disease classifications”, ICD-10 and ICD-O-3 are in fact used within EHR for recording diagnoses. The diagnosis is a way for the physician to describe the disease, which corresponds to an evolving process but, in fact, it is not the disease itself. A single disease may have multiple diagnoses all along its clinical course (for instance, an *in situ* neoplasm may evolve and become a malignant invasive neoplasm) but the disease (process) remains the same. Thus, when used in this context, disease classifications are, in fact, kinds of diagnoses which can be viewed as opinions about the undergoing disease. This assertion is in accordance with the definition proposed by Scheuermann et al. in [19] who claim that a **diagnosis** is a *“conclusion of an interpretive process that has as input a clinical picture of a given patient and as output an assertion to the effect that the patient has a disease of such and such a type. A diagnosis is a continuant entity that, once made, will survive through time, and is often supplanted by further diagnoses. The diagnostic process is thus iterative: the clinician is forming hypotheses during history taking, testing these during physical exam, forming new hypotheses as a result, and so on.”*

In the oncology field, a diagnosis describes two major facts about the disease: (i) the type of tumoral cells (*Morphology*) and (ii) its site of origin (*Topography*). Thus, ICD-10 and ICD-O-3 both allow to record diagnoses but their structure differs slightly. As a result, three different kinds of concepts are involved when considering these two terminologies:

- The morphology of the tumor, which is a representation of the pathological description of the tumor reported at a given time. Morphology is represented within the ICD-O-3 morphology axis.
- The topography of the tumor, which is a representation of the anatomical site of origin of the tumor reported at a given time. Topography is represented within the ICD-O-3 topography axis.
- The diagnosis, which is a representation of the reported description of the tumor and encompasses information about both the topography and the morphology of the tumor. Diagnosis is represented as such within ICD-10 and can be built by combining an ICD-O-3 topography and an ICD-O-3 morphology.

Because it is not possible to state that a *diagnosis* is equivalent to either a *topography* or a *morphology*, it is obviously not possible to find equivalences between concepts represented within these two terminologies. The unique correspondences that can be found between ICD-10 and ICD-O-3 concepts are thus a diagnosis (i.e., an ICD-10 code) mapped to a topography-morphology combination (i.e., a pair of an ICD-O-3 topography code and an ICD-O-3 morphology code).

### The national cancer institute thesaurus (NCIt)

*“NCI Thesaurus (NCIt) is NCI’s reference terminology. NCIt provides the concepts used in caCORE and caBIG to establish data semantics. It covers terminology for clinical care, translational and basic research, and public information and administrative activities. NCIt is also a widely recognized standard for biomedical coding and reference, used by a broad variety of public and private partners both nationally and internationally”* [20].

In the NCIt, topographies are described in the *Anatomic structure, system, or substance* axis. Morphologies and diagnoses are represented within the same hierarchy, subsumed by *Neoplasm*. Thus, no specific axis for tumor morphologies is defined and diagnoses are modeled as anatomic specializations of morphologies. For example, *Breast adenocarcinoma* *is_a* *Adenocarcinoma* is stated in:

$$\begin{aligned} \text{Breast adenocarcinoma} &\equiv \text{Adenocarcinoma}\\ &\cap \text{Breast carcinoma} \end{aligned} $$

Some NCIt concepts are annotated as being mapped to some ICD-O-3 morphologies. For example, *Invasive ductal carcinoma, not otherwise specified* is annotated as being mapped to two ICD-O-3 morphology codes (8500/3 *Infiltrating duct carcinoma, NOS* and 8521/3 *Infiltrating ductular carcinoma*). The semantics of this mapping annotation are not defined (i.e., exact match or another type of relationship). In the NCIt, even if the term *disease* is employed, it is not clear whether *Neoplasm* represents the disease or the diagnosis. For instance, in the NCI term Browser (https://ncit.nci.nih.gov/ncitbrowser/pages/home.jsf?version=16.10e), *Neoplasm* is defined as *“A benign or malignant tissue growth…”* and *“An abnormal mass of tissue…”*. Disease classifications are mainly used in EHR for diagnoses recording. In the remaining part of this manuscript, we use the NCIt concept *Neoplasm* as a kind of diagnosis describing the disease.

An OWL-DL representation of the NCIt is freely available in the Web ontology Language (OWL) format on the NCI website (https://cbiit.nci.nih.gov/evs-download/thesaurus-downloads). Although logic-based reasoning can be made with this OWL-DL representation, some inconsistencies have been identified and it has been shown that the NCIt should be used cautiously for this purpose [16–18].

### NCI Metathesaurus [21]

The NCI Metathesaurus (NCIm) is a biomedical terminology database *“that covers most terminologies used by NCI for clinical care, translational and basic research, and public information and administrative activities”* [21], including ICD-10 and ICD-O-3. It has been built and is maintained by the NCI. Its structure and a significant part of its concepts is based on the UMLS Metathesaurus [22]. Inside the NCIm, elements coming from different terminologies but representing the same biomedical notion are grouped into the same Concept Unique Identifier (CUI).

## Methods

**Fig. 1:**
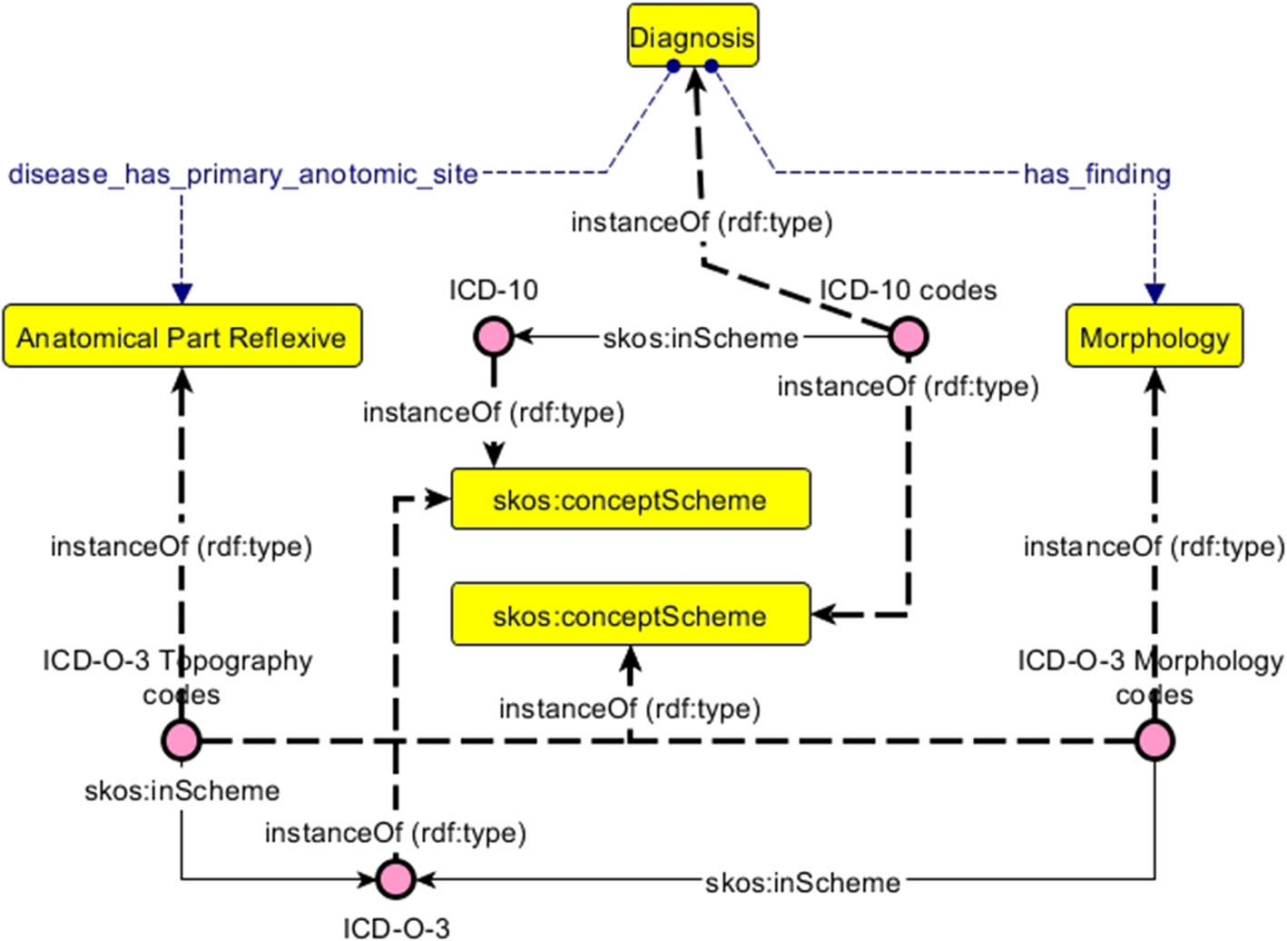
Graffoo [25] representation of the proposed model. The model is formal and semi-formal hybrid. Terminologies (ICD-10 and ICD-O-3) are represented in SKOS. Above them, a formal model is represented in OWL. Every OWL class of the formal model are subclasses of skos:Concept so that they can be instanciated by terminological artifacts

We focused our study on primary tumor descriptions, ignoring metastases and uncertain behaviors. ICD-10 and ICD-O-3 do not have a formal representation. In [23], authors recommend to use SKOS to describe the knowledge of such resources. In order to bridge these two terminologies, it is necessary to identify how concepts that are represented within them (diagnosis, morphology, topography) are related. These relationships should therefore be represented at the conceptual level so that they could be machine readable. Moreover, concepts represented by terminologies should be conceptually defined and related to corresponding codes. As a result, the targeted model remains independent from terminologies to be integrated, thus enabling the integration of other disease classifications. Our approach was to follow the W3C recommendations to define formal and semi-formal hybrid models [24] in order to build a model combining SKOS for the description of terminologies and OWL for representing involved concepts and for defining relationships between these concepts, as proposed in [23]. Figure 1 presents the organization of the proposed model using Graffoo [25].

The methods are composed of three steps:

- Defining a formal pattern for linking diagnosis, topography and morphology
- Building a model based on the NCIt corresponding to the formal pattern
- Instantiating the model with terminologies

### Defining a formal pattern for linking diagnosis, topography and morphology

In order to link ICD-10 and ICD-O-3 concepts, it is necessary to determine which relationships are involved and how these relationships associate concepts with each other. A topography-morphology combination in ICD-O-3 leads to a diagnosis description. ICD-O-3 axes can be viewed as descriptors that, when combined, provide necessary and sufficient information to represent a diagnosis. For instance, the diagnosis *Malignant neoplasm of lower-outer quadrant of breast* in ICD-10 can be defined as a malignant neoplasm arising from the lower-outer quadrant of breast (because it is defined as a presumed or stated primary malignant tumor within ICD-10). As stated above, the topography of a tumor (and more precisely, its primary site) is the anatomical site from which a tumor arises. As a result, a diagnosis has a specific relationship with the topography. The mention of “arising from” is ambiguous because this relationship implies that the tumor arises from the topography as a whole (lower-outer quadrant of breast) or from a part of this topography (a part of lower-outer quadrant of breast). Indeed, if a digestive system’s tumor is reported, it may refer to a tumor that originates from a part of the digestive system and not from the whole digestive system. In order to capture the fact that a primary tumor refers to a primary site as a whole and all its parts, we need specific topography classes. In [26], the W3C describes a way to represent those reflexive parts (e.g., *“Class(CarPart_reflexive complete unionOf(Car CarPart))”*). This pattern (called S-node) has been proposed for the biomedical domain in [27, 28]. Formally, we can define the *Malignant neoplasm of the lower-outer quadrant of breast* as a diagnosis whose morphology is a malignant neoplasm and whose primary site is the reflexive part of the lower-outer quadrant of breast. For describing the link between a *diagnosis* and its *morphology* as well as its *anatomical site*, we need to introduce the two following relationships (object properties in OWL parliance):

- has_morphology: *for modeling the relation between a diagnosis and the type of cells (morphology) that are stated to be involved in the tumor described by the diagnosis.*
- has_primary_site: *for modeling the relation between a diagnosis and an anatomical site (topography) that is stated to be the origin of the tumor described by the diagnosis.*

In description logics, the definition of the *Malignant neoplasm of the lower-outer quadrant of breast* diagnosis can be stated as follows:

- Malignant neoplasm of lower outer quadrant of breast ≡
  - Diagnosis
  - $\cap \exists $ has_morphology.Malignant neoplasm
  - $\cap \exists $ has_primary_site.Lower outer quadrant of breast Reflexive part

In addition, because of its expressivity, ICD-O-3 provides finer-grained information about the morphology of diagnoses than ICD-10 does. For instance, an adenocarcinoma arising from the lower-outer quadrant of breast can be reported using ICD-O-3. In ICD-10, there is no code corresponding to this diagnosis. However, an adenocarcinoma being a type of malignant neoplasm, an adenocarcinoma arising from the lower-outer quadrant of breast can be defined as a type of malignant neoplasm arising from the lower outer quadrant of breast (which is a coarser grained concept that exists in ICD-10). Formally, this can be expressed in description logics as follows:

-
  - (Diagnosis
  - $\cap \exists $ has_morphology.Adenocarcinoma
  - $\cap \exists $ has_primary_site.Lower outer quadrant of breast Reflexive part) $\subseteq $
- Malignant neoplasm of lower outer quadrant of breast

### Building a model based on the NCIt corresponding to the formal pattern

#### Building a part-whole lattice

In order to address the integration of diagnoses (ICD-10) with topographies and morphologies (ICD-O-3), the NCIt relationship *disease_has_primary_anatomic_site* is of particular interest. The NCIt’s definition of this relationship is: *“A role used to relate a disease to the anatomical site where the originating pathological process is located. The domain and the range for this role are ‘Disease, Disorder or Finding’ and ‘Anatomic Structure, System, or Substance”’*. This relationship is equivalent to the *has_primary_site* relationship defined in the previous subsection. As discussed on page 21, we consider that the primary anatomic site of a tumor encompasses the site itself and all its parts (this definition is in accordance with “is located”, which is mentioned in the NCIt definition of the *disease_has_primary_anatomic_site* relationship). In order to make this description possible, we have built a subsumption lattice composed of classes defined as the reflexive part of each *Anatomic Structure, System, or Substance*. For instance, the *Lower outer quadrant of breast Reflexive part* was defined as follows:

- Lower outer quadrant of breast Reflexive part ≡
  - Lower outer quadrant of breast
  - $ \cup \exists $ part_of.Lower outer quadrant of breast

The lattice was built with DL-reasoning over classes defined using two part-whole relationships available in the NCIt (namely *anatomic_structure_is_physical_part_of* and *anatomic_structure_has_location*).

#### Isolating morphologies

In contrast, no morphology axis is distinguished as such within the NCIt, and it is not possible to find a relationship equivalent to the aforementioned *has_morphology*. However, the NCIt provides a mapping between diagnoses and ICD-O-3 morphology codes. We have added classes corresponding to ICD-O-3 morphologies as types of the NCIt concept *Findings* and, based on the NCIt mappings, we have built new “refined” diagnosis concepts defined according to the following model:

- [NCIt diagnosis concept (refined)] ≡
  - [NCIt concept]
  - $ \cap \exists $ disease_has_finding.[Morphology mapped to the NCIt concept]

For instance, in the NCIt, *Adenocarcinoma* is mapped to the 8140/3 ICD-O-3 morphology (*Adenocarcinoma, NOS*) and we defined the corresponding “refined” NCIt concept as follows:

- Adenocarcinoma (refined) ≡
  - Adenocarcinoma
  - $ \cap \exists $ disease_has_finding.Adenocarinoma,NOS (ICD-O-3 morhology)

Each of the morphologies were classified depending on their tumoral behavior as described in ICD-O-3 (i.e., benign, malignant primary, in situ, malignant metastatic, unknown whether benign or malignant, unknown whether primary or metastatic).

#### Building the model

Using reflexive part anatomic concepts and morphologies and adapting the description logics’ expressions proposed in page 21, we have defined a formal pattern to describe relationships between diagnoses, morphologies and topographies within the “derivative” NCIt. In description logics, the pattern is the following:

- Diagnosis ≡
  - ∃ disease_has_finding.Morphology
  - $\cap \exists $ disease_has_primary_anatomic_site.Topography_Reflexive_Part

Based on the defined pattern, we implemented and executed the following algorithm:



Finally, we have implemented the model presented in Fig. 1 containing:

1. Morphologies,
2. Reflexive part topographies,
3. Diagnoses identified by the aforementioned algorithm.

#### Instantiating the model with disease classifications

**ICD-O3** ICD-O3 morphologies were represented as instances of the built-in morphology classes. ICD-O-3 topographies were represented as instances of the built-in reflexive part topographies. Reflexive part topographies to be instantiated by ICD-O-3 topographies were identified as follows:

1. Identify mappings between ICD-O-3 topographies and NCIt concepts having the same CUI within the NCIm,
2. Define these codes as instances of the corresponding NCIt concept,
3. Retrieve the corresponding reflexive part topography after DL-reasoning.

**ICD-10** ICD-10 codes were represented as instances of built-in diagnoses classes. Diagnoses to be instantiated by ICD-10 codes were identified as follows:

1. Identify mappings between ICD-10 codes and NCIt concepts having the same CUI within the NCIm,
2. Define concepts corresponding to ICD-10 codes based on the NCIt definition (by adding a restriction for the primary site based on the NCIt concept formal definition) and ICD-10 (by adding a restriction for the behavior) semantics. For instance :
*Breast, Unspecified* (C50.9) is a malignant primary neoplasm within the ICD-10 classification.
*Breast, Unspecified* (C50.9) has the same CUI as *Malignant Breast Neoplasm* (C9335) within the NCIm.
*Malignant Breast Neoplasm* (C9335) has as associated primary site *Breast* (C12971) within the NCIt.
  The built expression describing *Breast, Unspecified* (C50.9) was then:
  Malignant Breast Neoplasm
$\cap \exists $ disease_has_finding.Malignant primary neoplasm
$\cap \exists $ disease_has_primary_anatomic_site.Breast
3. Retrieve the corresponding diagnosis after DL-reasoning.

### Evaluation of the model

The National Cancer Institute provides, within the Surveillance, Epidemiology, and End Results (SEER) Program, a set of tools for ICD conversions [29]. We used the 2014-05-08 conversion file of ICD-O-3 to ICD-9-CM, to ICD-10 (Causes of Death) and to ICD-10-CM (available at http://seer.cancer.gov/tools/conversion/) as a gold standard for evaluating how ICD-O-3 and ICD-10 could be related. Based on this file, we have rebuilt ICD-O-3 topography-morphology combinations mapped to ICD-10 codes. A 2-step evaluation was then performed:

- For each combination, we queried the proposed model in order to evaluate how many branches of the diagnosis lattice were instantiated by both the ICD-10 code and the topography-morphology combination.
- We tried to build mappings based on the proposed model with a simple algorithm (ICD-10 codes and topography-morphology combinations with the minimum hierarchical edge-based distance were considered as mapped) and compared it with the gold standard.

## Results

All the analyses were processed over the OWL-DL version of the NCIt (14.11d) available at http://evs.nci.nih.gov/ftp1/NCI_Thesaurus/.

### Built model based on the NCIt

A total of 6720 topographies involved in at least one diagnosis definition was identified and the corresponding topographies reflexive parts were introduced in the NCIt in order to build the topography reflexive parts lattice (Section: “Building a part-whole lattice”). A total of 1120 NCIt codes was identified as being related to 1094 ICD-O-3 morphology codes. The 1094 corresponding morphology classes were added to the model and automatically classified under six general morphology classes depending on their behavior leading to a set of 1100 possible morphologies. Combining the 1100 morphology classes with the 6720 reflexive part topographies, 7392000 expressions were built. A total of 20133 (0.27%) expressions subsuming at least one NCIt code were identified and the corresponding classes were introduced in the model as diagnoses.

### Instantiating the model with disease classifications

Table 1 presents the part of each terminology that was covered by the final model. The numbers of codes to be integrated were:

- 409 ICD-O-3 topographies

**Table 1:** Part of ICD-O-3 and ICD-10 terminologies integrated within the final model

	Total number	Instantiating the final model
ICD-O-3 Topographies	409	278 (68.0%)
ICD-O-3 Morphologies	873	860 (98.5%)
ICD-10	727	302 (41.5%)
ICD-10 Benign	180	73 (40.5%)
ICD-10 In situ	66	22 (33.3%)
ICD-10 Malignant	481	207 (43.0%)
- 873 ICD-O-3 morphologies (excluding /6 *Malignant neoplasms, stated or presumed to be secondary* and /1 *Neoplasms of uncertain and unknown behavior*)
- 727 ICD-10 neoplasms (excluding C81-C96 *Malignant neoplasms, stated or presumed to be secondary* and D37-D48 *Neoplasms of uncertain or unknown behavior*)

Using the NCIm, 298 ICD-O-3 topography codes were linked to 540 NCIt codes. Within these NCIt codes, 29 were not subclasses of *Anatomic Structure, System, or Substance*. Among the 298 ICD-O-3 topography codes, 20 were related only to these 29 codes and were then excluded (e.g., C05.1 *Soft palate, NOS* was erroneously mapped to *Malignant Soft Palate Neoplasm*). Thus, 278 topography codes were finally included within the model as instances of the corresponding NCIt codes and classified as instances of topography reflexive parts after DL-reasoning. Using the NCIm, 302 ICD-10 codes were linked to NCIt codes. Building the corresponding expressions and after DL-reasoning, we were able to add 302 ICD-10 codes as instances of 380 diagnoses.

### Characteristics of the final model

The resulting model is constituted of 113643 axioms, including 27953 classes (6720 topographies, 1100 morphologies and 20133 diagnoses). A total of 1440 codes were instantiated (278 ICD-O-3 topographies, 860 ICD-O-3 morphologies and 302 ICD-10 codes).

Within the model, a significant part of ICD-10 (51%) and ICD-O-3 topography codes (28%) are instances of multiple classes (Table 2). This situation arises when the hierarchy of diagnoses within the NCIt is not in accordance with the topography or the morphology that we used to describe them. For example *Colon Cavernous Hemangioma* is a direct subclass of the following expressions:

**Table 2:** Number of codes instantiating multiple classes in the model

	N	Instances of multiple classes
ICD-O-3 Topographies	278	79 (28.4%)
ICD-O-3 Morphologies	860	0 (- %)
ICD-10	302	153 (50.7%)
ICD-10 Benign	73	26 (35.6%)
ICD-10 In situ	22	22 (100%)
ICD-10 Malignant	207	105 (50.7%)

- ∃ disease_has_finding.Cavernous hemangioma
  ∩∃ disease_has_primary_anatomic_site.Colorectal Region Reflexive part
- ∃ disease_has_finding.Cavernous hemangioma
  ∩∃ disease_has_primary_anatomic_site.Colon Reflexive part
- ∃ disease_has_finding.Hemangioma,NOS
  ∩∃ disease_has_primary_anatomic_site.Colorectal Region Reflexive part
- ∃ disease_has_finding.Hemangioma,NOS
  ∩∃ disease_has_primary_anatomic_site.Colon Reflexive part

The explanation for this situation is twofold: (1) there is neither an *anatomic_structure_has_location* relationship, nor an *anatomic_structure_is_physical_part_of* relationship between *Colon* and *Colorectal Region* within the NCIt; (2) *Colon Cavernous Hemangioma* is described as having these two anatomic structures as a primary site. On the other hand, through the NCIt diagnosis lattice, *Colon Cavernous Hemangioma* is described as being a subclass of the concepts *Cavernous hemangioma* and *Hemangioma, NOS*.

### Comparison with the SEER conversion file

Based on the SEER conversion file, excluding metastatic and uncertain behaviors from ICD-10 and ICD-O-3 morphologies, we were able to build 103950 mappings between an ICD-10 code and an ICD-O-3 topography-morphology combination. Due to the absence of some correspondences within the NCIm between the NCIt and ICD10 and between the NCIt and ICD-O-3, some ICD-10 and ICD-O-3 codes do not instantiate any class in the “derivative” model. As a result, 59% of these mappings could not be evaluated (because the ICD-10 code, the ICD-O-3 topography or the ICD-O-3 morphology was missing). Table 3 presents the results of the evaluation over the 42260 mappings combining codes which instantiate classes within the resulting model. The model relates 100% of the mappings through at least a diagnosis. A significant part of these mappings (36%) are related to more than one branch of the diagnosis lattice, especially for hematopoietic tumors (70%). Using the simple algorithm described above, the model was able to identify 42% of the mappings of the SEER file (61% for solid tumors and 5% for hematopoietic tumors). A quarter of these topography-morphology combinations were also mapped to another ICD-10 code, which is not consistent with the SEER file.

**Table 3:** Comparison with the SEER conversion program according to the tumor type (hematopoietic and solid tumors) and the number of branches of the diagnosis lattice that are identified for an ICD-10 code / ICD-O-3 combination

	All	Hematopoietic tumors	Solid tumors
	*N*=42260 (%)	*N*=14213 (%)	*N*=28047 (%)
Related in the model*	42260 (100.0)	14213 (100.0)	28047 (100.0)
More than 1 branch ^*a*^	15234 (36.1)	9910 (69.7)	5324 (18.9)
Mappings rebuilt from the model**	17766 (42.0)	739 (5.2)	17027 (60.7)
Non unique mappings ^*b*^	4886 (27.5)	333 (45.1)	4553 (26.7)

^*^Related in the model means that there is at least a common diagnosis inside the model that is instantiated by both the ICD-10 code and the ICD-O-3 combination

^**^Mappings rebuilt from the model corresponds to the mappings that we were able to rebuild automatically from the model

^*a*^More than 1 branch means that there is more than one branch of the diagnosis lattice that was instantiated by both the ICD-10 code and the ICD-O-3 combination

^*b*^Non unique mappings means that the topography-morphology combination was also mapped to another ICD-10 code (inconsistent with the SEER file)

## Discussion

### Implemented methods to build the model

We achieved to automatically build a model based on the NCIt, describing topographies, morphologies and diagnoses that can be instantiated by both ICD-O-3 and ICD-10 codes. As no morphological axis is available within the NCIt, we have adapted the NCIt by adding concepts corresponding to ICD-O-3 morphologies, which we related to the corresponding diagnoses (based on the ICD-O-3 annotation of the NCIt).

For the description of topographies, we have built an organ reflexive part lattice that enables the description of a primary site as encompassing the site itself and all its parts. These reflexive parts have been proposed for the biomedical domain in [27].

The diagnoses lattice was then automatically generated by DL-reasonners based on the topography and morphology lattices avoiding is_a overloading [17]. As a result, the obtained diagnoses subsumption lattice is a valid formal representation of diagnoses hierarchy in respect to the definitions of classes contributing to their description

In order to instantiate the model, we have used the NCIm to identify links between the NCIt and the terminologies (namely, ICD-10 and the ICD-O-3 topography axis). For ICD-10, we had to add a restriction based on the semantics available within the ICD-10 classification in order to ensure that primary tumors were described according to a primary site. The resulting model could not be instantiated completely by ICD-10 and ICD-O-3 codes for different reasons:

- The NCIt completeness for describing diagnoses. As our method relies on NCIt diagnoses, the resulting classes which were built depend on their existence within the NCIt (e.g., C00.1 *External Lower Lip malignant neoplasm* is not available within the NCIt).
- The NCIm provides a way to identify common concepts using the CUI but it can be incomplete or wrong (e.g., 20 ICD-O-3 topographies were mapped erroneously to non-anatomic concepts).

However, when the codes were found, we were able to identify a common diagnosis for all cases described in the SEER conversion program and, using a simple algorithm, 42% of the SEER mappings (corresponding to codes instantiating the model) could be rebuilt from the model. Our aim is not to enable conversion between codes but to provide a machine usable and semantically integrated view over them. From this perspective, the model is consistent because it provides links between ICD-10 diagnoses and ICD-O-3 topography-morphology combinations when they exist within the SEER conversion file. Moreover, the model describes many more possible relationships between diagnoses than the SEER conversion program does. For instance, in the latter, there is no relationship between *Adenocarcinoma, NOS – Colon, NOS* (C18.9 – M8140/3) and *Malignant neoplasm of rectosigmoid junction* (C19.9) whereas our model identifies successfully that they are both instances of *Malignant, primary site - Large Intestine Reflexive part*.

### Choice of the NCIt

In the biomedical field, other description logics-based terminologies exist. Specifically, SNOMED-CT^®;^ provides not only topography, morphology and diagnosis dimensions but also implements relationships between these concepts. However, the NCIt is specific to the oncology field and provides useful knowledge related to neoplasm diagnoses. The NCIt is freely and easily accessible.In contrast, SNOMED-CT has a much more restrictive affiliate license agreement and it is not easily accessible for countries which are not members of the International Health Terminology Standards Development Organisation (IHTSDO). In addition, it has been shown that SNOMED-CT’s formal representation suffers from the same flaws [30, 31] as the NCIt and has to be used cautiously while needing logic-based reasoning. Thus, a similar evaluation could be carried out on SNOMED-CT in order to estimate whether it could be useful for integrating disease classifications in oncology and to compare the results with what was found when using the NCIt.

### Limitations of the NCIt for integration purposes

In [18], Schultz et al. discussed that the OWL-DL version of the NCIt may lead to unexpected results which were not visible due to the lack of use cases needing logic-based reasoning over the OWL-DL version of the NCIt. The integration of heterogeneous disease classifications corresponds to such a use case. We have identified some limitations due to inconsistencies.

On the one hand, the NCIt provides concepts describing cancer diagnoses and, on the other hand, concepts describing the tumor topography. It also provides relationships which are involved in topography-morphology combinations, themselves expected to be equivalences of diagnoses. Its formal representation and the availability of an OWL version enable reasoning and the implementation of DL-queries. However, some intrinsic characteristics prevent its direct use for the integration of cancer disease classifications: (i) the absence of distinction between morphologies and diagnoses; (ii) diagnosis concepts described as having a specific primary site but not its parts. We have proposed a method to address these issues and to automatically build a consistent model based on the NCIt and the intrinsic semantics available within ICD-O-3 and ICD-10.

The obtained model representing diagnosis was classified using DL-reasoning ensuring concistency of the subsumption lattice. Linking these derivative consistent classes with diagnosis as represented within the NCIt can be used as an auditing tool. During the building process, a significant part of NCIt concepts was retrieved as subclasses of multiple diagnosis classes. As a result, the corresponding ICD-10 codes were defined as instances of multiple diagnosis classes and 36% of SEER mappings evaluated were retrieved as being related to more than one branch in the diagnosis lattice. For instance, the ICD-10 code C18.0 *Malignant neoplasm: Caecum* was mapped to the NCIt concept C9329 *Malignant Cecum Neoplasm*, which is related to multiple anatomic sites: *Gastrointestinal System*, *Cecum*, *Colon*, *Intestine* and *Colorectal Region*. As there is no relationship between *Cecum*, *Colorectal Region* and *Colon* within the NCIt (except that they are part of the *large intestine*), C18.0 instantiates the following classes:

- *Malignant, primary site – Cecum Reflexive part*
- *Malignant, primary site – Colon Reflexive part*
- *Malignant, primary site – Colorectal Region Reflexive part*

Two issues can be identified: (i) *Malignant Cecum Neoplasm* should not have *Colon* as an associated anatomic site within the NCIt because *Cecum* is neither a part, nor a subclass of *Colon*, (ii) *Cecum* and *Colon* should be related to *Colorectal Region*. The former is due to is_a overloading and has been discussed in [17]. The latter issue is due to the lack of part_of relationships within the NCIt. Another important issue can be identified for in situ neoplasms. 100% of the in situ ICD-10 codes are instances of more than one diagnosis within the model. The NCIt asserts that a *Carcinoma In situ* is a *Carcinoma*, which seems to be true. However, in the NCIt, *Carcinoma* is related to the *Carcinoma, NOS* ICD-O-3 morphology (having an invasive behavior) and *Carcinoma In situ* is related to the *Intraepithelial carcinoma, NOS* ICD-O-3 morphology (having an in situ behavior). Consequently, the subsumption relationship between *Carcinoma In situ* and *Carcinoma* is not consistent because a tumor cannot be both invasive and in situ at the same time. For instance, D05 *Carcinoma in situ of breast* is mapped to the NCIt concept C3641 *Stage 0 Breast Cancer*, which is related to the *Intraepithelial carcinoma, NOS* and *Epithelioma, NOS* ICD-O-3 concepts through the NCIt lattice. As a result, D05 instantiates the following classes:

- *Intraepithelial carcinoma, NOS - Breast Reflexive part*
- *Epithelioma, NOS - Breast Reflexive part*

Because *Intraepithelial carcinoma, NOS* has an in situ behavior and *Epithelioma, NOS* has a malignant, invasive behavior, it is not consistent to be an instance of these two diagnoses. This issue emphasizes erroneous mappings that may exist between ICD-O-3 and the NCIt due to ambiguous labels. A simple solution to this problem would be to add a concept representing the “Carcinoma” category of which both *Carcinoma* a *Carcinoma In situ* should be subclasses.

It is noteworthy that these patterns, which are mainly due to is_a overloading, can easily be retrieved by searching for codes which are instances of multiple diagnoses. By linking ICD-O-3 and ICD-10 terminologies to the NCIt and adding some restrictions based on their own semantics, our method may provide a useful auditing solution. Identifying those codes within the resulting model may enable discovery within the NCIt of: (i) structural inconsistencies (e.g., *Malignant cecum neoplasm* related to *Colon*), (ii) missing concepts (e.g., Carcinoma invasive that can be related to the ICD-O-3 concept Carcinoma, NOS) and (iii) missing relationships between concepts (e.g., *Cecum* which should be defined as a part of *Colorectal Region*).

In order to build the organ reflexive part lattice, parts of anatomical concepts were identified using transitive part-whole properties available within the NCIt (namely *anatomic_structure_is_physical_part_of* and *anatomic_structure_has_location*). This results in including cell parts as (indirect) subclasses of topographies (e.g. *Birbeck Granule* part_of *Langerhans Cell* part_of *Epidermis* part_of *Skin*). This would suggest that we allow a neoplasm to have *Birkbeck Granule* as primary site. Since the range of the disease_has_primary_anatomic_site property includes cells parts, such an assertion is allowed in the NCIt. Thereby, the built hierarchy is in accordance with the NICt representation of primary sites. As discussed in [28], the transitivity of the part_of property remains controversial. For instance, in [32], Rescher stated that *“A part (i.e., a biological sub-unit) of a cell is not said to be a part of the organ of which that cell is a part”*, which is in contradiction with that stated within the NCIt. However, diagnoses retained in the “derivative” model where those subsuming at least an NCIt concept so that diagnosis definitions remain realistic (because NCIt describes only existing, even if sometimes rare, tumors). Nevertheless, further work should be done in order to address this issue. In this context, patterns proposed by Schulz and Hahn in [28] are to be investigated.

### Perspectives

The NCIt is known to contain some inconsistencies. Thus, the OWL-DL version of the NCIt should be used cautiously. However, this resource is helpful in order to build a formal model for integrating heterogeneous cancer disease classifications. Indeed, the NCIt remains a rich knowledge resource and, as shown in this work, it is possible to extract parts of this resource and reorganize them so as to correct some of these inconsistencies (such as is_a overloading). Even if classes introduced in the “derivative” model are consistent (they have been classified based on their formal definition), instantiating them with terminology codes can lead to misclassifications. Our approach for classes instantiation is based on the classification of NCIt concepts within the “derivative” model, which in turn depends on the formal definition of NCIt concepts. We have proposed an approach, which identified possible misclassifications thanks to multiple classes instantiation by a single code. While this approach enables to find inconsistencies, there is a need for methods capable of selecting the class that should be instantiated ultimately in this situation.

Using the NCIm CUI to map the NCIt to ICD-O-3 and ICD-10 can be useful but is not enough because mappings are missing and some are inconsistent. We are currently working on a method based on the NCIm to identify additional mappings.

SNOMED-CT^®;^ exposes comparable structural characteristics with diagnosis, anatomic and even morphological concepts as well as relationships between them. Future work will explore SNOMED-CT as a resource for integration purpose. As SNOMED-CT is known to have the same inconsistencies as NCIt, we will study the feasibility of using both SNOMED-CT and the NCIt to build a consistent model addressing semantic and structural heterogeneities between disease classifications in oncology.

Topographies representation needs to be refined in order to avoid inconsistencies and define consistent levels of granularity for the propagation of the *disease_has_primary_anatomic_site* property. The Foundational Model of Anatomy (FMA) Ontology [33] *“is a domain ontology that represents a coherent body of explicit declarative knowledge about human anatomy”* [34]. Further work will explore the ability to define these topographies based on the FMA.

The main goal of this work is to provide a consistent resource for the integration of heterogeneous disease classifications in oncology. While our approach based on the NCIt seems promising, two main limitations have been discussed (misclassification of codes within the “derivative” model and incomplete coverage due to NCIm mapping methods). The proposed pattern for the integration of disease classifications in oncology enables to extract knowledge from available resources. We will apply the same approach to other resources (e.g., SNOMED-CT and FMA) so as to enrich the “derivative” model. This future work will allow a better coverage and we will take advantage of existing links between concepts within these resources (i.e., anatomical descriptions from the FMA) and between resources (i.e., mappings available within the NCIm).

In addition, based on this “derivative” model, algorithms for disease identification can be built. This resource can manage heterogeneity by providing an integrated view of diagnoses recorded in EHRs. As a result, algorithms based on classes of the “derivative” model can use transparently available data coded with disease classifications and focus on building consistent rules for disease identification.

## Conclusion

We have proposed a method to automatically build a model for integrating ICD-10 and ICD-O-3 based on the NCIt. The resulting “derivative” model is a consistent machine understandable resource that enables an integrated view of these heterogeneous terminologies. The NCIt structure and the available relationships can help to bridge disease classifications taking into account their structural and granular heterogeneity. However, (i) inconsistencies exist within the NCIt leading to misclassifications when instantiating the “derivative” model with terminologies, (ii) the “derivative” model only integrates a part of ICD-10 and ICD-O-3. The NCit is not sufficient for integration purpose and further work based on other termino-ontological resources is needed in order to enrich the model and avoid identified inconsistencies.

## Additional file

Additional file 1 The “derivative model” is available at: https://github.com/vianneyJouhet/ModelTerminologyIntegrationOncology/tree/master/data. (OWL 31932 kb)
